# Negative Associations of Stress and Anxiety Levels With Cytotoxic and Regulatory Natural Killer Cell Frequency in Chronic Tinnitus

**DOI:** 10.3389/fpsyg.2022.871822

**Published:** 2022-06-23

**Authors:** Laura Basso, Benjamin Boecking, Patrick Neff, Petra Brueggemann, Linda El-Ahmad, Jelena Brasanac, Matthias Rose, Stefan M. Gold, Birgit Mazurek

**Affiliations:** ^1^Tinnitus Center, Charité – Universitätsmedizin Berlin, Berlin, Germany; ^2^Center for Cognitive Neuroscience and Department of Psychology, University of Salzburg, Salzburg, Austria; ^3^Department of Psychiatry and Psychotherapy, University of Regensburg, Regensburg, Germany; ^4^Center for Neuroprosthetics, Institute of Bioengineering, École Polytechnique Fédérale de Lausanne, Geneva, Switzerland; ^5^Department of Radiology and Medical Informatics, University of Geneva, Geneva, Switzerland; ^6^Medical Department, Section of Psychosomatic Medicine, Charité – Universitätsmedizin Berlin, Berlin, Germany; ^7^Department of Psychiatry and Psychotherapy, Charité – Universitätsmedizin Berlin, Berlin, Germany; ^8^Institute of Neuroimmunology and Multiple Sclerosis (INIMS), Universitätsklinikum Hamburg-Eppendorf, Hamburg, Germany

**Keywords:** tinnitus, stress, natural killer cells, depression, anxiety, inflammation, immune phenotyping

## Abstract

**Background:**

Depression and anxiety are known to be associated with stress-induced changes in the immune system. Bothersome tinnitus can be related to stress and often co-occurs with depression and anxiety. This study investigates associations of psychological and audiological tinnitus-related factors with inflammatory parameters and immune cell subsets in chronic tinnitus patients as well as treatment-related effects.

**Methods:**

This longitudinal study of inpatients treated with compact multimodal tinnitus-specific cognitive behavioral therapy included four repeated measurement sessions: baseline (*N* = 41), treatment end, 7.8-week (*N* = 35), and 13.8-week follow-up (*N* = 34). Data collection included audiometric testing, blood sampling, and psychometric questionnaires: Tinnitus Handicap Inventory (THI), Perceived Stress Questionnaire (PSQ-20), and Hospital Anxiety Depression Scale (HADS). Flow cytometry was used to analyze immune cell subsets. Statistical analyses comprised correlation and network analysis (cross-sectional), and linear mixed effect models (longitudinal).

**Results:**

Bootstrapped network analysis showed negative averaged cross-sectional associations of cytotoxic natural killer (NKc) cell frequency (CD56 + CD16+) and PSQ-20 (−0.21 [−0.48, 0]) and of regulatory natural killer (NKreg) cell frequency (CD56 + CD16dim/−) and HADS anxiety (−0.14 [−0.38, 0]). No significant treatment effects were found. A negative predictive effect of baseline PSQ-20 scores (*β* = −6.22 [−12.18, −0.26], *p* = 0.041) and a positive predictive effect of baseline ferritin levels (*β* = 8.90 [2.76, 15.03], *p* = 0.004) on NKc cell frequency across the repeated measurement sessions were observed.

**Conclusion:**

We observed negative relationships between perceived stress levels and NKc cell frequency and between anxiety levels and NKreg cell frequency in chronic tinnitus patients. These exploratory results suggest stress−/anxiety-related immune alterations in bothersome tinnitus but need to be tested in further confirmatory studies with larger sample sizes. The potential of NK cells as biomarkers of emotional distress in chronic tinnitus should be further investigated.

## Introduction

Psychological stress, both acute and chronic, is known to influence the immune system (Segerstrom and Miller, 2004). Chronic stress-induced inflammation appears to play an important role in both anxiety and mood disorders (Salim et al., 2012; Slavich and Irwin, 2014; Wohleb et al., 2015; Otte et al., 2016; Michopoulos et al., 2017). Major depressive disorder (MDD) is not only characterized by stress-mediated alterations of the immune system but the interplay between innate and adaptive immunity and neuroendocrine circuits may be implicated in its pathophysiology (Haapakoski et al., 2016).

Numerous alterations in the peripheral immune system have been described in patients with MDD, including increased pro-inflammatory cytokine levels, namely interleukin(IL)-6 and tumor necrosis factor(TNF)-α (Dowlati et al., 2010), and reduced lymphocyte proliferation and decreased natural killer (NK) cell cytotoxicity (Zorrilla et al., 2001). Moreover, several findings indicate altered frequencies of immune cells subsets in depression, including a shift in the monocyte phenotype (Hasselmann et al., 2018; Lynall et al., 2020), decreased percentages of NK cells (Suzuki et al., 2017; Patas et al., 2018; Schiweck et al., 2020), increased percentages of helper T cells (Lynall et al., 2020; Schiweck et al., 2020), increased or decreased percentages of regulatory T cells (Li et al., 2010; Grosse et al., 2016; Suzuki et al., 2017; Patas et al., 2018), and increased percentages of B cells (Schiweck et al., 2020). For other stress-associated disorders such as posttraumatic stress disorder (PTSD), similar immune alterations have been observed with increased pro-inflammatory cytokine levels and altered immune cell distributions (Michopoulos et al., 2017).

Tinnitus often occurs in combination with stress-related psychological disorders, with prevalence rates of 33% for depression (Salazar et al., 2019) and 45% for anxiety disorders (Pattyn et al., 2016). Psychological factors seem to be associated with both the presence and severity of chronic tinnitus (Trevis et al., 2018) and the impact of tinnitus on quality of life can be reduced by cognitive behavioral therapy (CBT; Fuller et al., 2020). The role of (chronic) stress appears to be particularly important in bothersome tinnitus (Hébert et al., 2017; Mazurek et al., 2019; Elarbed et al., 2021), i.e., tinnitus that is associated with suffering and emotional distress.

Immunological disturbances in tinnitus (Szczepek and Mazurek, 2017; Haider et al., 2021; Kang et al., 2021) might represent a possible link between bothersome tinnitus and depression/anxiety. Immune alterations in chronic tinnitus have not yet been studied extensively, but some previous findings include increased inflammatory parameters such as neutrophil-to-lymphocyte ratio (NLR; Ozbay et al., 2015; Yildiz et al., 2020; Demir, 2021) and positive associations between tinnitus-related distress and TNF-α (Szczepek et al., 2014). Furthermore, stress-mediated immune alterations in tinnitus could potentially be positively influenced by psychological treatment. This is suggested by a study in which a reduction in TNF-α levels and a concomitant reduction in tinnitus disturbance, perceived stress levels, anxious depression, and anger symptoms were observed after a 10-week relaxation program in chronic tinnitus patients (Weber et al., 2002).

The present study aims to evaluate stress-mediated changes in inflammatory parameters and immune cell subsets in chronic tinnitus by investigating their association with psychological and audiological tinnitus-related factors. Moreover, this study aims to assess possible treatment-related changes in relevant inflammatory and immune indices by tinnitus-specific CBT. This might provide insights into the effectiveness of CBT-based treatment to improve potential immunological alterations in chronic tinnitus. Participants were investigated before compact multimodal tinnitus-specific CBT, directly after, and at a planned 6- and 12-week follow-up to assess cross-sectional associations as well as treatment-related changes in psychological symptoms and immunological/inflammatory parameters. Overall, we expect to find associations of immune cell subsets or inflammatory parameters with measures of emotional distress in chronic tinnitus (tinnitus-related distress, perceived stress levels, and/or anxiety and depression levels) and to observe treatment-related changes in the psychological status and identified immunological biomarkers.

## Materials and Methods

### Sample

In total, 41 participants with chronic subjective tinnitus (for at least 3 months) were recruited for this study. Participants were in-patients receiving treatment at the Tinnitus Center between July 2019 and March 2020, consisting of a short-term multimodal CBT-based treatment program specifically designed for chronic tinnitus lasting 4 to 5 days (*M* = 4.59, SD = 0.5). The treatment included ENT and general medical examinations, education, counseling, individual and group CBT sessions, auditory attention training, relaxation, and physiotherapy (Basso et al., 2022a). The recruitment of new participants was stopped in spring 2020 due to the beginning of the COVID-19 pandemic; the completion of follow-up measurement sessions lasted until August 2020. Inclusion criteria were chronic subjective tinnitus, age ≥ 18 years, and written informed consent; exclusion criteria were inability to consent due to serious mental or physical impairments, simultaneous participation in other research studies, pronounced hearing deterioration/sudden hearing loss in the last 4 to 6 weeks, and known autoimmune diseases. Around two-thirds of the sample were male (*N* = 26, 63.41%); on average, participants were 52.05 years old (SD = 10), ranging from 26 to 67 years. Most participants had bilateral tinnitus (*N* = 30, 73.17%) and normal hearing (*N* = 26, 63.41%). Sample characteristics are summarized in Table 1. The study was approved by the local ethics committee (Charité–Universitätsmedizin Berlin, EA1/055/19), and all participants provided written informed consent before enrolment.

**Table 1:** Sample description including sociodemographic factors, tinnitus−/hearing-related factors, health−/lifestyle-related factors, and psychometric questionnaires (*N* = 41).

**Variable**		***M***	**SD**	***N***	**%**
**Sociodemographic factors**					
Sex	Female			15	36.59
	Male			26	63.41
Age (years)		52.05	10.00	41	
Marital status	Single			9	21.95
	Married/living together			24	58.54
	Separated/divorced/widowed			8	19.51
Education^a^	Low			13	31.71
	Intermediate			15	36.59
	High			13	31.71
Employed				29	70.73
**Tinnitus/Hearing**					
Tinnitus type	Intermittent			15	36.59
	Constant			26	63.41
Tinnitus localization	Left			9	21.95
	Right			2	4.88
	Bilateral			30	73.17
Tinnitus loudness (dB SL)		20.22	14.12	32	
Tinnitus frequency (Hz)		5804.69	2131.50	32	
Hearing threshold (dB)^b^		24.21	13.64	41	
	No impairment (≤25)			26	63.41
	Mild impairment (26–40)			12	29.27
	Moderate impairment (41–60)			2	4.88
	Severe impairment (61–80)			1	2.44
	Profound impairment (≥81)			0	0.00
Hearing aid user				10	24.39
Loudness discomfort level (dB)	Average	75.66	14.62	41	
	Minimum	65.55	15.29	41	
	Maximum	86.28	13.23	41	
ABR wave I latency (ms)		1.65	0.13	34	
ABR wave III latency (ms)		3.85	0.22	40	
ABR wave V latency (ms)		5.78	0.26	40	
**Health/Lifestyle**					
Smoking				6	14.63
Cigarettes smoked per week		8.29	34.03	41	
Drinking alcohol				17	41.46
Alcohol units consumed per week^c^		1.83	3.43	41	
BMI (kg/m^2^)^d^		25.59	3.22	41	
	Underweight (<18.50)			0	0.00
	Normal (18.50–24.99)			21	51.22
	Overweight (25–29.99)			15	36.59
	Obese (≥30)			5	12.20
Systolic blood pressure (mmHg)		125.39	16.35	41	
Diastolic blood pressure (mmHg)		78.56	17.56	41	
Food intake prior to sampling (g)		84.32	70.19	41	
Beverage intake prior to sampling (ml)		471.71	303.76	41	
Medication					
	Antidepressants			7	17.07
	Antihypertensives			12	29.27
	Lipid-lowering drugs			2	4.88
	Pain medication			9	21.95
	Other			24	58.54
**Psychometric Questionnaires**					
THI: Tinnitus handicap		38.25	24.74	40	
	Slight (0–16)			8	20.00
	Mild (18–36)			15	37.50
	Moderate (38–56)			6	15.00
	Severe (58–76)			8	20.00
	Catastrophic (78–100)			3	7.50
PSQ-20: Perceived stress		45.50	22.47	40	
	Normal (≤ 50)			25	62.50
	Mild (51–66)			9	22.50
	Moderate (67–83)			5	12.50
	Severe (≥ 84)			1	2.50
HADS: Anxiety		7.65	3.81	40	
	Normal (0–7)			20	50.00
	Mild (8–10)			11	27.50
	Moderate (11–14)			7	17.50
	Severe (15–21)			2	5.00
HADS: Depression		6.10	4.61	40	
	Normal (0–7)			27	67.50
	Mild (8–10)			5	12.50
	Moderate (11–14)			5	12.50
	Severe (15–21)			3	7.50

ABR, Auditory Brainstem Response; BMI, Body-Mass-Index; HADS, Hospital Anxiety and Depression Scale; PSQ-20, Perceived Stress Questionnaire (20-item version); THI, Tinnitus Handicap Inventory

a Education levels: low = elementary, secondary or middle school; medium = high school or completed apprenticeship; high = university.

b Mean hearing threshold over all measured frequencies. Grading of hearing thresholds: World Health Organization (1991).

c One unit = 0.3 l beer or 0.2 l wine or shot glass of spirits.

d BMI classification: World Health Organization (2000).

### Design

**Figure 1:**
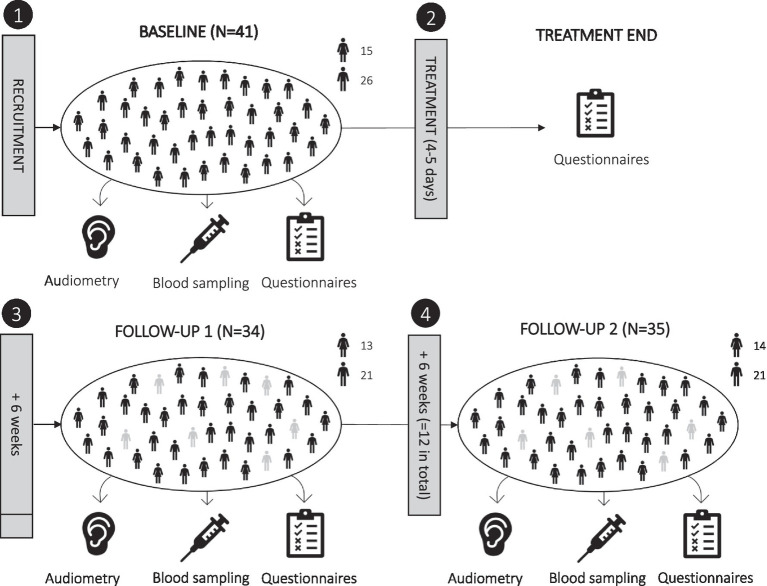
Overview of study design. The study included four scheduled measurement sessions: (1) baseline, (2) directly after treatment (4–5 days later), (3) 6-week follow-up, and (4) 12-week follow-up. Actual follow-ups were performed on average after 7.8 and 13.8 weeks. Dropouts are shown in grey.

This exploratory longitudinal study included four planned measurement sessions: baseline, directly after treatment, a 6-week follow-up, and a 12-week follow-up; see Figure 1. Baseline data collection was performed on the morning of treatment begin and included blood sampling, psychometric questionnaires, and the following audiometric tests: pure tone audiometry (PTA), tinnitus pitch and loudness matching, loudness discomfort level (LDL), and auditory brain stem response (ABR; which was measured 1 day later). Directly after treatment (4–5 days later), only psychometric questionnaire data were collected. The first follow-up session was planned after 6 weeks; on average, it took place 7.79 weeks (SD = 3.13) after treatment and included audiometric testing (PTA and tinnitus matching), blood sampling, and psychometric questionnaires. The second and last follow-up session was planned after 12 weeks; on average, it was performed 13.77 weeks (SD = 3.65) after treatment and included audiometric testing (PTA, tinnitus matching, LDL, ABR), blood sampling, and psychometric questionnaires.

At baseline, *N* = 41 participants were included; *N* = 33 of which completed both follow-up measurement sessions (*N* = 2 missed only the first follow-up, *N* = 1 missed only the second follow-up, *N* = 5 missed both the first and second follow-up). Reasons for dropping out were illness/hospitalization (*N* = 5), moving away (*N* = 1), or the effort associated with the study (*N* = 2). Of the *N* = 33 participants who completed both follow-ups, blood collection at the second follow-up was incomplete for one participant, and blood samples for immunophenotyping were missing for *N* = 6 participants. This resulted in a sample size of *N* = 26 for immune cell subset data and of *N* = 32 for most other parameters across the repeated measurement sessions.

### Audiometric Testing

All audiometric tests were performed at the audiological department of the clinic in sound-proof booths. Hearing aid users were asked to remove their devices before all tests. PTA was performed for the following frequencies: 0.25, 0.5, 1, 1.5, 2, 3, 4, 6, and 8 kHz; in case of high-frequency tinnitus, also for 10 kHz (*N* = 1 at baseline and follow-up 2). Hearing thresholds were recorded in 5-decibel (dB) intervals for each ear, and the mean hearing threshold was calculated across all measured frequencies and both sides. LDL was measured using pure tones for the same frequencies as the hearing thresholds in each ear individually. LDL values were averaged across all measured frequencies and both sides for statistical analyses.

The tinnitus pitch and loudness matching procedure (using pure tones or narrow-band noise) was first performed for pitch/frequency (Hz), then for loudness (dB), as described previously (Basso et al., 2022b). Before matching, patients were asked (1) whether tinnitus was currently audible, (2) whether it was perceived on the left, right or both sides, (3) whether it sounded more like pure tones or noise, and (4) whether its frequency was low, medium or high (Basso et al., 2022b). The starting point for frequency matching was the specified frequency range (low, medium, or high) with sounds presented approx. 10 dB above the respective hearing threshold; then, after successful frequency matching, loudness was adjusted in 1-dB steps starting at the hearing threshold (Basso et al., 2022b). Final matches for both frequency and loudness had to be confirmed twice by the patients (Basso et al., 2022b). Mean frequency and loudness values across both sides were calculated for bilateral tinnitus. Tinnitus loudness in dB sensation level (SL) was determined for analysis (i.e., tinnitus loudness adjusted for hearing threshold). Tinnitus matching was not possible in eight cases at baseline and in six cases at the follow-ups, either because tinnitus was not currently audible (intermittent tinnitus), had a different sound quality than pure tones or narrow-band noise, or the tinnitus frequency was above 10 kHz.

ABR recordings were obtained in the standard clinical setup of the audiological department including two different ABR systems: Eclipse (Interacoustics, Denmark) and Corona (Pilot Blankenfelde, Germany). Both used a click stimulus (alternating) with an intensity level of 80 dB nHL. Each ear was tested individually. ABR amplitude peaks were determined by visual inspection. For statistical analyses, absolute wave I, wave III, and wave V latencies (ms) were averaged across both sides. Amplitudes were not included because they were not routinely documented.

### Blood Sampling and Biomarker Quantification

All blood samples were collected in the morning between 9 and 11 am to control for circadian rhythms: mean sampling times were 10.10 am (SD = 16 min.) at baseline, 09.59 am (SD = 38 min.) at follow-up 1, and 09.42 am (SD = 35 min.) at follow-up 2. Blood pressure was always measured in addition to blood sampling. In total, 103.5 ml of blood was collected per session. Some samples were analyzed at a clinically licensed diagnostic lab (Labor Berlin – Charité Vivantes GmbH) for full blood count and the quantification of other parameters, while the rest was transferred to the neuropsychiatry laboratory (Department of Psychiatry and Psychotherapy, Charité – Universitätsmedizin Berlin) for processing and storage. There, peripheral blood mononuclear cells (PBMCs) were isolated using density-gradient centrifugation and established standard operating procedures (Hasselmann et al., 2018). Blood was first diluted in phosphate-buffered saline (PBS; 1:1), then 35 ml of diluted blood was carefully layered on top of 15 ml of Biocoll density medium (Biochrome, Germany) in a 50-ml conical tube and centrifugated at 870 × *g* for 30 min. (brakes off). The mononuclear cell layer from the interphase was collected and washed two times for 10 min. in cold PBS. Pelleted PBMCs were resuspended in RPMI-1640 (Gibco, ThermoFisher Scientific, Germany) supplemented with 25% heat-inactivated fetal bovine serum (FBS; Biochrome, Germany) and 10% dimethylsulfoxide (Applichem GmbH, Germany) for cryopreservation. Cells were counted and placed in 1.5 ml tubes (Eppendorf, Germany) at the concentration of 10 million cells/ml. Cells were first stored in Mr. Frosty freezing container (Sigma-Aldrich, United States) for slow overnight cooling in a − 80°C freezer and transferred the next day to a long-term liquid nitrogen storage tank (−196°C) where they stayed until further analysis.

#### Full Blood Count and Quantification of Other Blood Parameters

Laboratory tests included full blood count and the following other parameters: fibrinogen, high-density lipoprotein (HDL) cholesterol, low-density lipoprotein (LDL) cholesterol, ferritin, C-reactive protein (CRP), IL-1β, IL-6, TNF-α, vascular endothelial growth factor A (VEGF-A), insulin-like growth factor-I (IGF-1). The neutrophil-to-lymphocyte ratio (NLR) and platelet-to-lymphocyte ratio (PLR) were calculated based on absolute values. Summary statistics of the quantified blood parameters (at baseline) with classifications based on adult reference ranges utilized by the laboratory can be found in Table 2.

**Table 2:** Summary statistics of blood parameters at baseline (*N* = 41).

**Parameter**	***N***	**%**	***M***	**SD**	**Min**	**Max**
Fibrinogen (g/l)	41		2.95	0.54	1.86	4.49
Increased		4.88				
HDL cholesterol (mg/dl)	39		59.18	15.10	25.00	97.00
Decreased		5.13				
LDL cholesterol (mg/dl)	39		123.41	29.85	53.00	193.00
Increased		35.90				
Ferritin (*μ*g/l)	39		169.81	122.14	7.70	542.20
Decreased		2.56				
Increased		12.82				
C-reactive protein (mg/l)	39		1.31	1.40	0.30	8.70
Increased		2.56				
Interleukin-1b (pg/ml)	41		5.96	3.42	5	24
Increased		14.63				
Interleukin-6 (ng/l)	37		1.68	0.33	1.50	2.90
Tumor necrosis factor-α (pg/ml)	41		5.70	1.69	4.00	11.30
Vascular endothelial growth factor-A (pg/ml)	41		173.15	89.22	31	349
Increased		41.46				
Insulin-like growth factor-1 (ng/ml)	41		142.59	44.17	59.00	261.90
Increased		4.88				
IGF-1 standard deviation score	40		0.67	0.92	−1.03	3.01
Leukocytes (/nl)	41		6.65	1.68	4.07	10.97
Increased		2.44				
Erythrocytes (/pl)	41		4.71	0.47	3.80	5.80
Decreased		7.32				
Haemoglobin (g/dl)	41		14.23	1.35	12.00	17.80
Decreased		7.32				
Increased		4.88				
Haematocrit (l/l)	41		0.41	0.04	0.33	0.51
Decreased		14.63				
Increased		2.44				
MCV (fl)	41		87.59	4.01	75	99
Decreased		2.44				
MCH (pg)	41		30.30	1.51	25.30	32.90
Decreased		2.44				
MCHC (g/dl)	41		34.63	1.12	32.20	37.20
Increased		9.76				
RDW-CV (%)	41		12.77	0.77	11.30	15.10
Decreased		2.44				
Increased		2.44				
Platelets (/nl)	41		256.56	44.72	169	344
MPV (fl)	41		10.93	0.84	8.90	13.10
Neutrophils: absolute (/nl)	41		4.19	1.47	1.93	8.12
Increased		2.44				
Neutrophils: %	41		61.93	8.97	40	77
Decreased		2.44				
Increased		2.44				
Immature Granulocytes: absolute (/nl)	41		0.02	0.02	0.01	0.14
Increased		2.44				
Immature Granulocytes: %	41		0.33	0.20	0.20	1.40
Increased		2.44				
Lymphocytes: absolute (/nl)	41		1.74	0.48	0.88	3.28
Decreased		4.88				
Lymphocytes: %	41		27.06	7.69	14.20	46.30
Decreased		17.07				
Increased		2.44				
Monocytes: absolute (/nl)	41		0.54	0.17	0.29	0.99
Increased		4.88				
Monocytes: %	41		8.17	2.08	4.30	13.10
Increased		21.95				
Eosinophils: absolute (/nl)	41		0.12	0.10	0.00	0.46
Decreased		4.88				
Eosinophils: %	41		1.90	1.59	0.00	6.50
Decreased		12.20				
Increased		7.32				
Basophils: absolute (/nl)	41		0.05	0.05	0.01	0.31
Basophils: %	41		0.62	0.21	0.20	1.10
Neutrophil-to-lymphocyte ratio	41		2.57	1.07	0.87	5.47
Platelet-to-lymphocyte ratio	41		155.51	42.56	89.91	270.21
CD4 T cells (%)	24		71.30	10.69	45.40	87.50
CD8 T cells (%)	24		22.38	8.98	9.35	40.20
Cytotoxic natural killer cells (%)	26		66.23	13.65	43.50	94.10
Regulatory natural killer cells (%)	26		24.41	13.50	2.79	47.80
Classical monocytes (%)	26		82.73	9.67	46.80	92.70
Intermediate monocytes (%)	26		5.65	3.84	0.87	16.70
Non-classical monocytes (%)	26		2.73	2.76	0.60	13.10
B cells (%)	26		53.06	18.31	7.73	84.80
Dendritic cells (%)	26		35.55	13.42	9.29	68.50

*HDL, High-Density Lipoprotein; IGF-1, Insulin-Like Growth Factor-1; LDL, Low-Density Lipoprotein; MCH, Mean Corpuscular Hemoglobin; MCHC, Mean Corpuscular Hemoglobin Concentration; MCV, Mean Corpuscular Volume; MPV, Mean Platelet Volume; RDW-CV, Red Cell Distribution Width - Coefficient of Variation*.

#### Immune Phenotyping

Immune phenotyping by flow cytometry was performed on cryopreserved PBMCs as previously described (Hasselmann et al., 2018). A T cell panel, containing anti-CD3, -CD4, -CD8 was used to analyze CD4+ and CD8+ T cells and a non-T cell panel containing anti-CD14, -CD16, -CD20, -HLA-DR, -CD56, -CD4, and -CD3 antibodies to distinguish B cells, monocytes, natural killer cells, and dendritic cells. In the first step, PBMCs were incubated with a live/dead marker (Zombie NIR Fixable Viability Kit, BioLegend, United States) and the CCR7 antibody in PBS for 15 min. in the dark at room temperature. Second, antibody premixes were added in staining buffer (PBS + 2 mm EDTA Sigma-Aldrich, Germany +0.2% bovine serum albumin Miltenyi Biotec, Germany) and incubated for an additional 15 min. Lastly, cells were washed and resuspended in staining buffer and immediately acquired on a FACSCanto II (BD, Germany). All samples from the same individual (baseline, follow-up 1, follow-up 2) were analyzed in the same run on the same day to avoid any systematic effects due to technical variability. Frequencies of the following immune cell subsets were identified: CD4+ and CD8+ T cells; cytotoxic natural killer cells (NKc; CD56 + CD16+) and regulatory natural killer cells (NKreg; CD56 + CD16dim/−); classical (CD14++CD16−), non-classical (CD14 + CD16++), and intermediate (CD14++CD16+) monocytes; B cells (CD20+); and dendritic cells (HLA-DR+). Immune phenotyping was performed for *N* = 26 participants (due to 6 missing samples). Moreover, two additional subjects had to be excluded from analysis in the T cell panel due to a genetic variation that interferes with CD45RA antibody binding. Summary statistics of immune cell subsets (at baseline) can be found in Table 2.

### Psychometric Questionnaires

German versions of the following psychometric questionnaires were used: Tinnitus Handicap Inventory (THI) consisting of 25 items (Kleinjung et al., 2007) to measure tinnitus-related distress; the 20-item version of the Perceived Stress Questionnaire (PSQ-20; Fliege et al., 2001, 2005) to measure the general perceived stress level; and the Hospital Anxiety and Depression Scale (HADS) consisting of 14 items (Herrmann-Lingen et al., 2011) to assess anxiety and depression levels.

### Statistical Analysis

Statistical analyses were performed using R (R Core Team, 2020) and included descriptive analyses, correlation analyses, network analyses, and t-tests (cross-sectional), as well as linear mixed-effects models (longitudinal). All analyses are described in more detail in the next sections. The significance level was set to *p* < 0.05.

#### Cross-Sectional Analyses

For descriptive analyses, sample size, mean and standard deviation or category frequencies for each variable are listed in Tables 1, 2. Reference values for blood parameters are based on laboratory specifications. To explore associations between lifestyle, psychological, and audiological factors with immune cell subsets and other blood parameters, nonparametric Spearman correlations were calculated and visualized [using *ggstatsplot* (Patil, 2021) and *ggcorrplot* (Kassambara, 2019)]. Network analysis was performed based on the results of correlation analyses using LASSO-regularized network estimation [using *qgraph* (Epskamp et al., 2012) and *bootnet* (Epskamp et al., 2018)] to investigate averaged cross-sectional interrelations between identified factors (correlated psychological/audiological and blood parameters and relevant control variables). Network estimation was based on averaged values across all repeated measurement sessions. For regularized network estimation (sparse Gaussian graphical model), graphical LASSO based on extended BIC criterion (EBICglasso) was used (Foygel and Drton, 2010; Epskamp and Fried, 2018). The tuning parameter gamma was set to 0.5 and a threshold was applied to increase specificity. In addition, 95% confidence intervals of edge-weights were estimated based on non-parametric bootstrapping (Epskamp et al., 2018) including 1,000 bootstrapped networks. All variables included in the bootstrapped network estimation were normally distributed (Kolmogorov–Smirnov test). In addition, to evaluate possible influences of medications on identified biomarkers, two-sample t-tests were calculated (assumptions were met) to examine whether baseline levels of identified biomarkers differed in patients using antidepressants, antihypertensives, lipid-lowering drugs, pain medication, or other medications.

#### Longitudinal Analyses

Six linear mixed-effects models [*lme4* (Bates et al., 2015)] with random intercept terms (subjects) were calculated for the prediction of change in psychometric questionnaires (THI, PSQ-20, HADS) and relevant biomarkers identified by cross-sectional analyses (NKc and NKreg cell frequencies) across all repeated measurement sessions (baseline, treatment end, follow-up 1, follow-up 2). Age (centered and scaled) and sex were included as covariates in all models. For the prediction of NK cell frequencies, additional predictor variables (centered and scaled) were included based on the cross-sectional results; for the change in NKc cell frequency: baseline ferritin levels, baseline PSQ-20 scores, and the interaction between baseline PSQ-20 scores and time; for the change in NKreg cell frequency: baseline HADS anxiety levels and their interaction with time. For THI scores, square root transformation (due to the presence of zero values) was used to achieve normally distributed residuals (Kolmogorov–Smirnov test); all other outcomes were not transformed. Models were fitted by REML and z-tests were used for significance testing [using *multcomp* (Hothorn et al., 2008)].

## Results

### Cross-Sectional Analysis

#### Sample Description

Sample characteristics including baseline sociodemographic factors, tinnitus−/hearing-related factors, health−/lifestyle-related factors, and psychometric questionnaire scores can be found in Table 1. All baseline blood parameters are summarized in Table 2.

#### Correlations Between Lifestyle Factors, Psychological Factors, Audiological Factors, Immune Cell Subsets, and Other Blood Parameters

**Figure 2:**
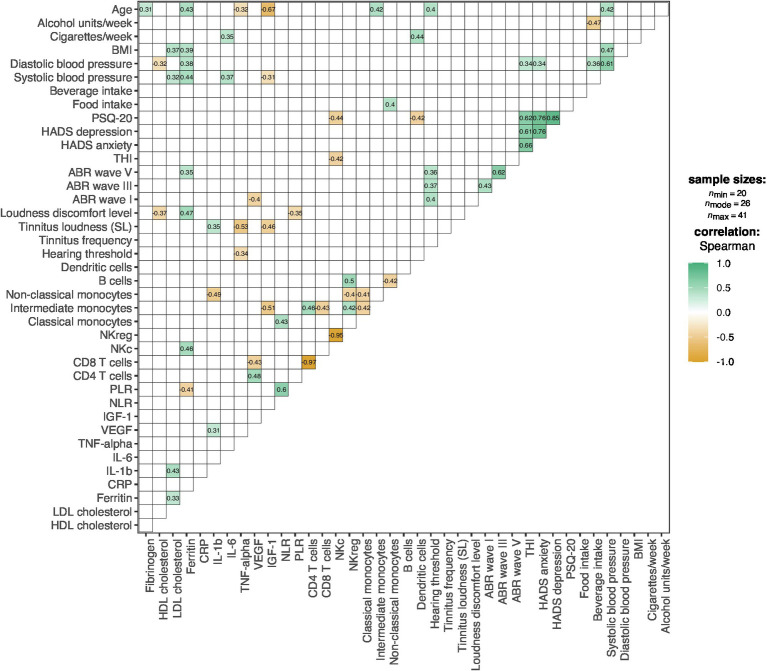
Correlation plot for the associations between lifestyle, psychological, audiological factors, immune cell subsets, and other blood parameters. Colors represent the direction and degree of significant pairwise correlations (Spearman); positive correlations are shown in green and negative correlations in orange; non-significant correlations (*p* > 0.05) are omitted. ABR, Auditory Brainstem Response; BMI, Body-Mass-Index; CRP, C-Reactive Protein; HADS, Hospital Anxiety and Depression Scale; HDL, High-Density Lipoprotein; IGF-1, Insulin-Like Growth Factor-I; IL, Interleukin; LDL, Low-Density Lipoprotein; NKc, Cytotoxic Natural Killer Cells; NKreg, Regulatory Natural Killer Cells; NLR, Neutrophil-to-Lymphocyte Ratio; PLR, Platelet-to-Lymphocyte Ratio; PSQ-20, Perceived Stress Questionnaire (20-item version); SL, Sensation Level; THI, Tinnitus Handicap Inventory; TNF-alpha, Tumor Necrosis Factor-alpha; VEGF-A, Vascular Endothelial Growth Factor A.

Correlations between lifestyle factors, psychological factors, audiological factors, immune cell subsets, and other blood parameters are shown in Figure 2. Significant correlations between psychological factors and immune cell subsets were observed for: THI scores and NKc cell frequency, *r* = −0.42, *p* = 0.037 (*n* = 25); PSQ-20 and NKc cell frequency, *r* = −0.44, *p* = 0.028 (*n* = 25); and PSQ-20 scores and dendritic cell frequency, *r* = −0.42, *p* = 0.039 (*n* = 25). No significant correlations between audiological factors and immune cell subsets were present.

Regarding other investigated blood parameters (fibrinogen, HDL cholesterol, LDL cholesterol, ferritin, CRP, IL-1b, IL-6, TNF-α, VEGF, IGF-1, NLR, PLR), no correlations with psychological factors were present. With audiological factors, correlations were found for: hearing threshold and TNF-α levels, *r* = −0.34, *p* = 0.028 (*n* = 41); tinnitus loudness (SL) and TNF-α levels, *r* = −0.53, *p* = 0.002 (*n* = 32); tinnitus loudness (SL) and IL-1b levels, *r* = 0.35, *p* = 0.047 (*n* = 32); tinnitus loudness (SL) and IGF-1 levels, *r* = −0.46, *p* = 0.008 (*n* = 32); loudness discomfort level and HDL cholesterol levels, *r* = −0.37, *p* = 0.019 (*n* = 39); loudness discomfort level and ferritin levels, *r* = 0.47, *p* = 0.002 (*n* = 39); loudness discomfort level and PLR, *r* = −0.35, *p* = 0.023 (*n* = 41); ABR wave I latency and VEGF levels; *r* = −0.40, *p* = 0.020 (*n* = 34); and ABR wave V latency and ferritin levels, *r* = 0.35, *p* = 0.030 (*n* = 38).

#### Network Analysis: Averaged Cross-Sectional Connections

The observed correlations were further analyzed by investigating the interrelations between the identified factors (averaged across baseline, follow-up 1, and follow-up 2) in two LASSO regularized networks. The first network included tinnitus loudness (SL) and correlated blood parameters (TNF-α, IL-1b, IGF-1) and their covariates (age, hearing threshold, systolic blood pressure, LDL cholesterol, VEGF, non-classical monocytes, intermediate monocytes). The second network included all correlated psychological factors and blood parameters (THI, PSQ-20, NKc cells, dendritic cells) and their covariates (HADS anxiety and depression, diastolic blood pressure, NKreg cells, ferritin, and smoking).

**Figure 3:**
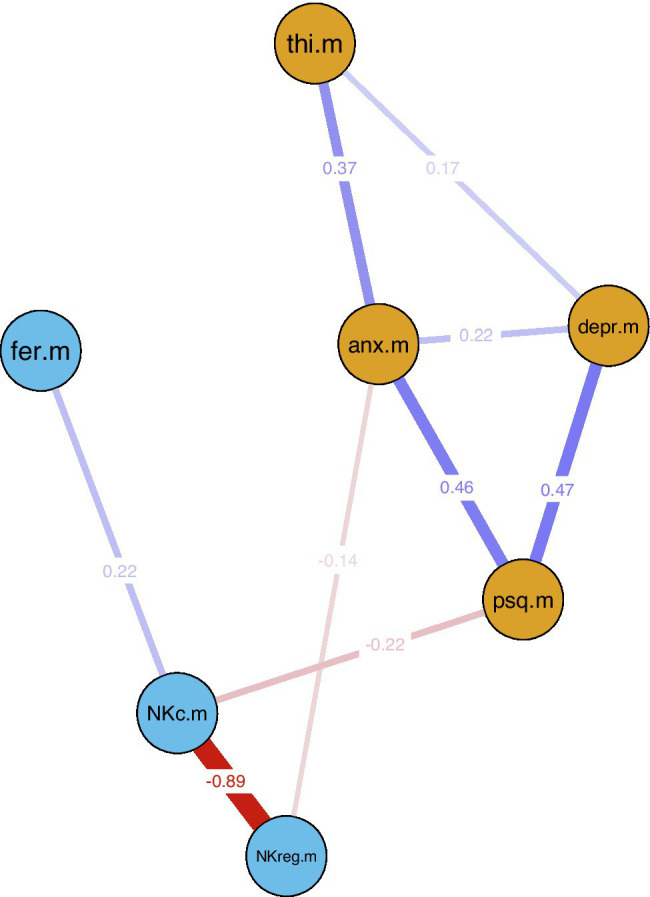
LASSO regularized averaged cross-sectional network estimation for identified associations between psychological factors and natural killer cells. Blue lines indicate positive and red lines negative associations; line width indicates association strength. Network estimation was based on averaged variable values across all repeated measurement sessions (baseline, 7.8-week follow-up, 13.8-week follow-up). Nodes: anx.m = mean HADS anxiety; depr.m = mean HADS depression; NKc.m = mean NKc frequency; NKreg.m = mean NKreg frequency; psq.m = mean PSQ-20 total score; thi.m = mean THI total score. HADS, Hospital Anxiety and Depression Scale; NKc, Cytotoxic Natural Killer Cells; NKreg, Regulatory Natural Killer Cells; PSQ-20, Perceived Stress Questionnaire (20-item version); THI, Tinnitus Handicap Inventory.

**Figure 4:**
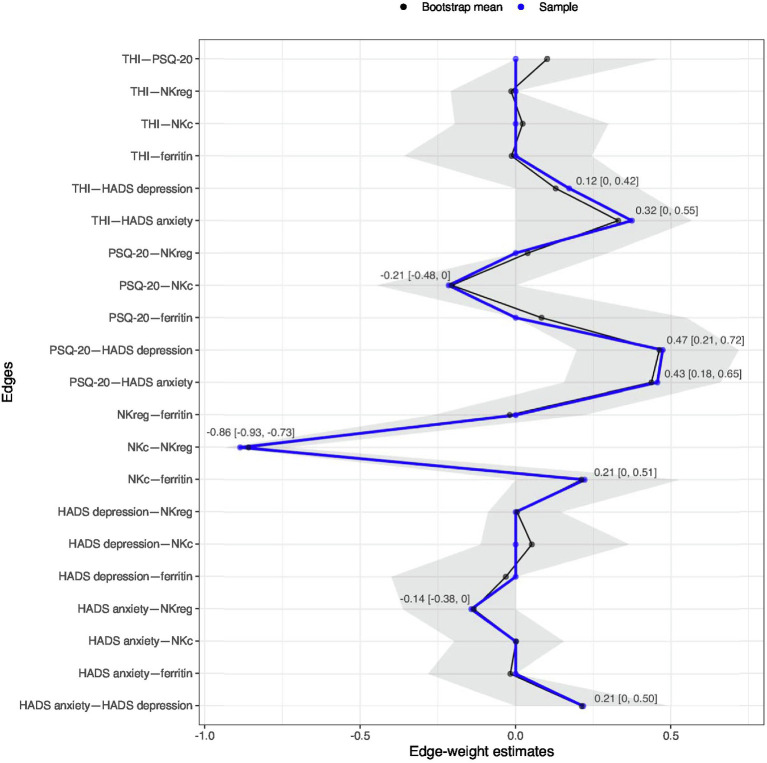
Estimated edge weights in the sample (blue line) and bootstrapped mean (black line) over 1,000 bootstrapped networks; grey area shows bootstrapped 95%-confidence intervals (CIs). Numbers in the plot indicate the bootstrapped mean [95%-CIs] for all edges observed in the original network. Network estimation was based on averaged variable values across all repeated measurement sessions (baseline, 7.8-week follow-up, 13.8-week follow-up). HADS, Hospital Anxiety and Depression Scale; NKc, Cytotoxic Natural Killer Cells; NKreg, Regulatory Natural Killer Cells; PSQ-20, Perceived Stress Questionnaire (20-item version); THI, Tinnitus Handicap Inventory.

In the first network, only one connection/edge was present (not shown): a positive association between age and IGF-1 (−0.45). No further analyses were performed for this network. For the second network, all variables without any connections to the other investigated factors in the first estimation were removed (dendritic cells, smoking, diastolic blood pressure), and the network was estimated again only with connected factors; see Figure 3. For this network, additional bootstrapping of confidence intervals (CIs) was performed. The following negative edges (sorted by strength of association) were present in the estimated network: mean NKc cell frequency and mean NKreg cell frequency; mean PSQ-20 scores and mean NKc cell frequency; mean HADS anxiety scores and mean NKreg cell frequency. The following positive edges (sorted by strength of association) were present: mean PSQ-20 and mean HADS depression scores; mean PSQ-20 and mean HADS anxiety scores; mean THI and mean HADS anxiety scores; mean NKc cell frequency and mean ferritin levels; mean HADS depression and mean HADS anxiety scores; and mean THI and mean HADS depression scores. Non-parametric bootstrapping to obtain the 95%-CIs included 1,000 bootstrapped networks; results are shown in Figure 4. Note: Bootstrapped CIs can be used to compare the accuracy of edge-weight estimates, but should not be used for significance testing of LASSO regularized edge-weights (Epskamp et al., 2018).

#### Influence of Medications

To examine the influence of medications on NK cell frequencies, two-sample t-tests were calculated. No significant (baseline) differences in NKc and NKreg cell frequencies were found for patients taking antidepressants (*N* = 7; NKc: *p* = 0.351; NKreg: *p* = 0.332), antihypertensives (*N* = 12; NKc: *p* = 0.250; NKreg: *p* = 0.286), pain medication (*N* = 9; NKc: *p* = 0.534; NKreg: *p* = 0.798), or other medications (*N* = 24; NKc: *p* = 0.359; NKreg: *p* = 0.647). For patients taking lipid-lowering drugs (*N* = 2), no test could be performed because NK cell frequencies were missing.

### Longitudinal Analysis

#### Change Across All Repeated Measurement Sessions

**Figure 5:**
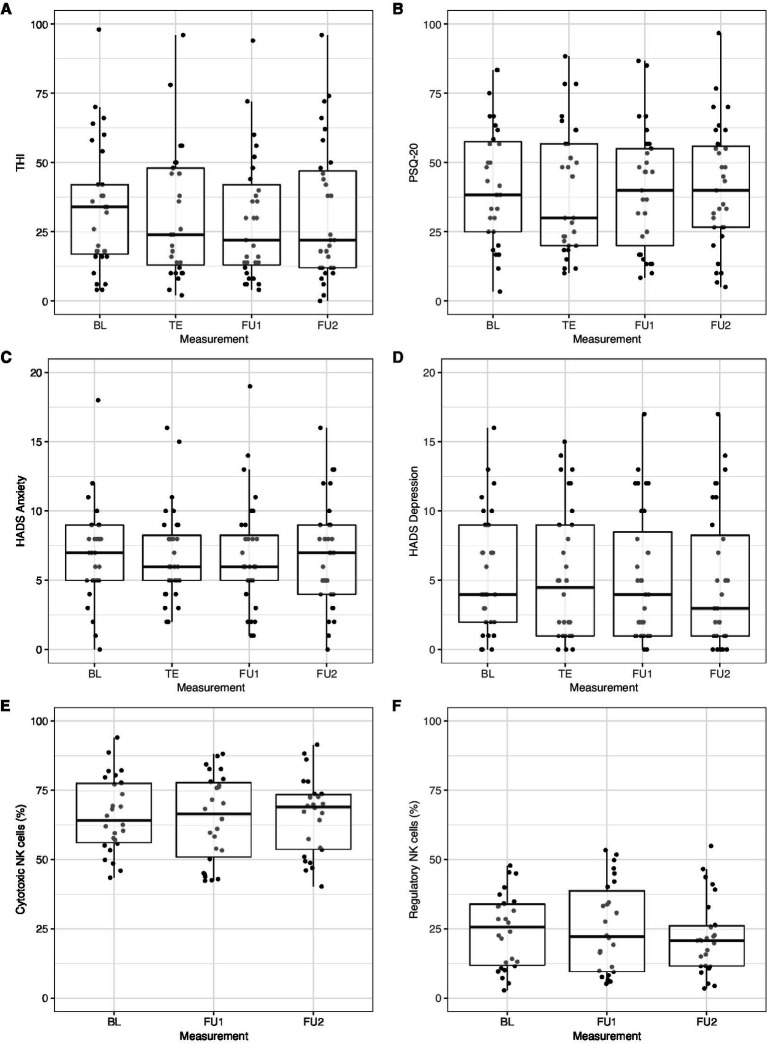
Change in **(A)** THI, **(B)** PSQ-20, **(C)** HADS anxiety, **(D)** HADS depression, **(E)** cytotoxic natural killer cell (NKc), and **(F)** regulatory natural killer cell (NKreg) frequency across measurement sessions. Only participants with complete data from all repeated measurement sessions were included: **(A)** THI and **(B)** PSQ-20: *n* = 31; **(C)** HADS anxiety and **(D)** HADS depression: *n* = 32; **(E)** NKc and **(F)** NKreg: *n* = 26. BL, Baseline; FU1, Follow-up 1 (7.8 weeks); FU2, Follow-up 2 (13.8 weeks); HADS, Hospital Anxiety and Depression Scale; PSQ-20, Perceived Stress Questionnaire (20-item version); TE, Treatment end; THI, Tinnitus Handicap Inventory.

Psychometric questionnaire scores (THI, PSQ-20, HADS) and NK cell frequencies (NKc and NKreg) across all repeated measurement sessions in participants with complete data are shown in Figures 5A–F.

Linear mixed-effects models with random subject intercepts and the control variables sex and age were calculated to test for changes in THI, PSQ-20, HADS anxiety, and HADS depression scores over time. These models revealed no significant changes in these psychological outcome variables across the repeated measurement sessions (THI: *p* = 0.061; PSQ-20: *p* = 0.810; HADS anxiety: *p* = 0.467; HADS depression: *p* = 0.113).

For the prediction of change in NK cell frequencies, baseline PSQ-20 scores and ferritin levels were included as predictors as well as the interaction between baseline PSQ-20 scores and time (in addition to sex and age). No change in NKc cell frequencies across measurement sessions was observed (*p* = 0.992) but a significant negative effect of baseline PSQ-20 scores, *β* = −6.22 [−12.18, −0.26], SE = 3.04, *z* = −2.05, *p* = 0.041, and a positive effect of baseline ferritin levels, *β* = 8.90 [2.76, 15.03], SE = 3.13, *z* = 2.84, *p* = 0.004, on NKc cell frequencies across all measurement sessions. The interaction between baseline PSQ-20 scores and time was not significant (*p* = 0.905).

For the prediction of change in NKreg cell frequencies, baseline HADS anxiety scores and their interaction with time were included as predictors (in addition to sex and age). No change in NKreg cell frequencies was observed across measurement sessions (*p* = 0.273), no effect of baseline HADS anxiety scores (*p* = 0.894), and no interaction between baseline HADS anxiety scores and time (*p* = 0.721).

## Discussion

This study investigated the associations of inflammatory parameters and the immune cell phenotype with tinnitus-related psychological and audiological factors in 41 participants with chronic tinnitus as well as potential treatment-related changes. Cross-sectional results (averaged LASSO regularized network analysis) showed negative relationships between perceived stress levels and the frequency of cytotoxic natural killer (NKc) cells (CD56 + CD16+) and between anxiety levels and the frequency of regulatory natural killer (NKreg) cells (CD56 + CD16dim/−). No effects of medications on NKc and NKreg cell frequencies were observed in our sample. The longitudinal analysis revealed no significant treatment-related changes in psychological measures (tinnitus-related distress, perceived stress, anxiety, or depression levels) or NK cell frequencies. There was a negative effect of baseline perceived stress levels and a positive effect of baseline ferritin levels on NKc cell frequency across the repeated measurement sessions.

Most NK cells exert a cytotoxic function, while some have a regulatory function in the immune system by releasing cytokines (Dragoş and Tănăsescu, 2010). NK cells are known to be strongly affected by stress (Dragoş and Tănăsescu, 2010; Capellino et al., 2020). Acute stress leads to an increased number of cytotoxic NK cells in the blood, whereas chronic stress is associated with a decrease in NK cell cytotoxic activity (Segerstrom and Miller, 2004; Dragoş and Tănăsescu, 2010). Reduced NK cell frequency (Grosse et al., 2016; Suzuki et al., 2017; Patas et al., 2018; Schiweck et al., 2020) and impaired NK cell function (Evans et al., 1992; Zorrilla et al., 2001) have been observed in depression. Similarly, impaired NK cell activity has been observed in PTSD (Pace and Heim, 2011). In addition, a recent meta-analysis on the effects of psychosocial interventions on immune system function showed that CBT was associated with increases in NK cell activity (Shields et al., 2020).

In the present study, effects were found for both cytotoxic and regulatory NK cell frequencies. For NKc cells, a negative association with perceived stress was found in correlation analysis, averaged cross-sectional network analysis, and longitudinal analysis, with perceived stress levels at baseline negatively predicting NKc cell frequency across the repeated measurements. No significant association with psychological factors was observed for NKreg cell frequency in the correlation and longitudinal analyses, but a negative relationship with anxiety levels was present in the averaged cross-sectional network analysis. Thus, the association between NKc cells and perceived stress levels appears to be more robust in our sample than the association between NKreg cells and anxiety levels. However, due to the exploratory nature of this study, both findings require further investigation. Of the few studies that examined immune changes in tinnitus, our results are partially in line with those of Savastano et al. (2007), who report a non-significant trend for a positive correlation between natural killer cells (CD16 + CD56NK) and daily satisfaction (psychological and physical functioning) in tinnitus patients (*p* = 0.032/0.023).

Despite the correlation between tinnitus-related distress and NKc cell frequency, there was no direct association between tinnitus-related distress and NK cell frequencies in the network analysis, only indirect associations *via* perceived stress levels and anxiety symptoms. Stress and anxiety are related to tinnitus severity, yet there is also a certain conceptual overlap between these constructs (Ooms et al., 2012; Trevis et al., 2018; Elarbed et al., 2021). In our sample, tinnitus-related distress, perceived stress levels, and anxiety/depression symptoms were strongly correlated (all correlations above *r* = 0.6, *p* < 0.001). We hypothesize that the observed associations of perceived stress levels and anxiety symptoms with NKc and NKreg cell frequencies represent effects of emotional distress in chronic tinnitus patients, indicative of general psychological rather than tinnitus-specific mechanisms. Because most participants in our sample had normal or mild perceived stress (85%) and anxiety (77.5%) levels, these effects were observed in the non-clinical range. This suggests that alterations in NK cell frequency in chronic tinnitus patients with emotional distress (stress/anxiety) might be present even in the absence of a fully developed mood or anxiety disorder.

Potentially, sleep disturbances might represent a link for the observed negative relationships between stress/anxiety levels and NK cell frequencies. Sleep disturbances are common in chronic tinnitus and appear associated with tinnitus-related emotional and cognitive distress (Crönlein et al., 2016). Moreover, sleep deprivation strongly affects the immune system, including NK cell number and activity (Irwin, 2002; van Leeuwen et al., 2009). In our sample, 65.9% (*N* = 27) reported (sometimes) having difficulties falling to sleep because of their tinnitus. Moreover, intrusive thoughts appear related to reduced NK cell cytotoxicity in healthy stressed individuals (Segerstrom and Miller, 2004). Therefore, tinnitus intrusiveness might constitute an important factor in this regard. However, tinnitus-related distress was measured by the THI, which includes sleep problems and tinnitus intrusiveness, and no direct effect of the THI on NK cell frequencies was observed in network analysis. The specific role of sleep disturbances and tinnitus intrusiveness on immunological changes in chronic tinnitus and their links to stress/anxiety could be important questions for further research.

Regarding treatment effects, we expected treatment-induced changes in tinnitus-related distress and psychological symptoms based on previous studies with the same or similar treatment interventions (Seydel et al., 2010, 2015; Brueggemann et al., 2018, 2019; Basso et al., 2022a), but no significant effects were observed. Neither stress and anxiety levels nor NK cell frequencies showed significant treatment-related changes. Overall, the lack of treatment effects may have been influenced by the small sample size and the short treatment duration. For perceived stress levels, an initial treatment-induced decline appears to have diminished over time, suggesting that the beneficial effect of the short-term treatment was not sustained over time. This may suggest that longer-term or repeated interventions are needed. The lack of significant improvement in psychological well-being likely explains the lack of changes in NK cell frequencies. The positive association of baseline ferritin levels with NKc cell frequency across the repeated measurements is in line with the observation of lower NK cell number in healthy female runners with lower ferritin concentrations (Flynn et al., 2003).

No consistent effects were found for the other blood parameters studied, partially in contrast to previous tinnitus research (see Haider et al., 2021; Kang et al., 2021). For the inflammatory markers IL-6, CRP, and NLR, no associations were found with psychological or audiological tinnitus-related variables. Previous studies have observed increased NLR in tinnitus patients (Ozbay et al., 2015; Yildiz et al., 2020; Demir, 2021), but conflicting findings exist as well (Bayram et al., 2016; Düzenli et al., 2018). While we observed correlations of IL-1b, TNF-α, and IGF-1 with tinnitus loudness, these associations did not persist in network analysis. This was particularly surprising for TNF-α. TNF-α concentrations are known to be increased after acute stress (Marsland et al., 2017), in depression (Dowlati et al., 2010) and anxiety disorders/PTSD (Renna et al., 2018; Yang and Jiang, 2020), and there is also evidence suggesting an involvement of TNF-α in noise-induced hearing loss and tinnitus (Wang et al., 2019; Shulman et al., 2021). In an exploratory study in 30 chronic tinnitus patients, a positive correlation between TNF-α and tinnitus loudness (determined by a visual analog scale) and a negative correlation with the subscale “joy” of the PSQ was observed (Szczepek et al., 2014). Weber et al. (2002) found a decrease in TNF-α levels in their sample of 26 chronic tinnitus patients after a 10-week relaxation program in addition to psychological symptom reduction. The lack of consistent results regarding TNF-α in the present study may have been influenced by the small sample size and the generally low levels of tinnitus-related distress and psychological symptoms in our sample.

With regard to other parameters, previous studies that examined lipid levels in tinnitus patients found higher total cholesterol levels (Martines et al., 2015; Avcı, 2021), higher LDL (Avcı, 2021), lower HDL (Ensari et al., 2019), and higher triglyceride levels (Ensari et al., 2019; Avcı, 2021) in tinnitus patients compared to controls. In our sample, 35.9% had increased LDL and 5.1% decreased HDL cholesterol levels compared to reference values. However, no direct control group was included in the present study.

Furthermore, in addition to NK cells, other immunophenotype changes in depressed patients have been reported in the literature, including monocytes (Hasselmann et al., 2018; Lynall et al., 2020), helper T cells (Lynall et al., 2020; Schiweck et al., 2020), regulatory T cells (Li et al., 2010; Grosse et al., 2016; Suzuki et al., 2017; Patas et al., 2018), and B cells (Schiweck et al., 2020). Beyond NK cells, no associations of immune cell subsets with psychological variables were found in our chronic tinnitus sample. It is possible that such immunophenotype changes are not present in tinnitus, only in tinnitus patients with higher emotional distress, or that respective associations could not be detected here due to the small sample size.

### Limitations

This study has several limitations. Because of the lack of a control group, no information could be obtained on whether inflammatory markers and the frequency of immune cell subsets are altered in tinnitus compared with healthy controls. The sample size of this study was relatively small and was further reduced by missing values for immune cell subsets and by dropouts in the repeated measurement sessions. This may have limited the power of our study, particularly with regard to the evaluation of treatment-related changes. Moreover, because of the exploratory nature of the study and the large number of variables investigated (40 variables in the correlation analysis) in a comparatively small sample (*N* = 41), no adjustment for multiple testing was applied. This may have increased the risk of obtaining false-positive results (type I error). Overall, these exploratory results should be interpreted with caution and need to be tested in further confirmatory studies.

### Conclusion

In this study, we observed negative relationships between perceived stress levels and NKc cell (CD56 + CD16+) frequency as well as between anxiety levels and NKreg cell (CD56 + CD16dim/−) frequency in chronic tinnitus. These results are consistent with the literature on mood and anxiety disorders, as reduced NK cell frequency or function is known to occur in stress-related psychological conditions. These results suggest that emotional distress (stress/anxiety) may negatively affect NK cell frequency in chronic tinnitus. This should be further investigated, also with respect to possible influences of sleep disturbances and tinnitus intrusiveness. A major limitation of the present study is the small sample size. Larger studies are needed to test the validity of these results and to further investigate the potential of NKc and NKreg cell frequencies as distress-related biomarkers in chronic tinnitus.

## Data Availability Statement

The datasets presented in this article are not readily available because no consent of the participants to publish their data was obtained. Requests to access the datasets should be directed to BM (birgit.mazurek@charite.de).

## Ethics Statement

The studies involving human participants were reviewed and approved by the local Ethics Committee of Charité – Universitätsmedizin Berlin (EA1/055/19). The patients/participants provided their written informed consent to participate in this study.

## Author Contributions

LB: conceptualization, project administration, investigation, formal analysis, visualization, and writing—original draft. BB: supervision and writing—review and editing. PN: conceptualization, methodology, supervision, and writing—review and editing. PB: writing—review and editing. LE-A: investigation. JB: investigation and writing—review and editing. MR: writing—review and editing. SG: conceptualization, resources, project administration, writing—review and editing. BM: conceptualization, supervision, funding acquisition, resources, project administration, and writing—review and editing. All authors contributed to the article and approved the submitted version.

## Funding

This project has received funding from the European Union’s Horizon 2020 research and innovation programme under the Marie Skłodowska-Curie grant agreement No 764604, and the Heinz und Heide Dürr Stiftung.

## Conflict of Interest

The authors declare that the research was conducted in the absence of any commercial or financial relationships that could be construed as a potential conflict of interest.

## Publisher’s Note

All claims expressed in this article are solely those of the authors and do not necessarily represent those of their affiliated organizations, or those of the publisher, the editors and the reviewers. Any product that may be evaluated in this article, or claim that may be made by its manufacturer, is not guaranteed or endorsed by the publisher.
